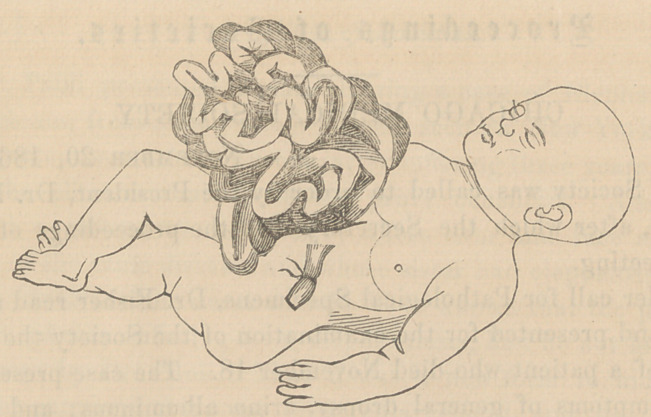# Ectropium Intestinorum

**Published:** 1869-01

**Authors:** Geo. Fredigke

**Affiliations:** Chicago


ARTICLE V.

### ECTROPIUM INTESTINORUM.

By GEO. FREDIGKE, M.D., Chicago.

*History.*—The individual affected was a boy, born at 6.30 A. M., the 15th November, by Mrs. B., wife of Jacob B., and attended by Mrs. F., a midwife. The mother is 21 years old, healthy, and was delivered of a girl 16 months ago, so that this was her second child.

*Description.*—From an opening 2 inches in length at the umbilical region, ¼ inch to the right of the umbilicus, and parallel to the linea alba, bulged out the greater portion of the intestines. Their coats were hypertrophied, and the abdominal wall was to such an extent contracted as to allow only the admission of the small finger on both sides of the orifice. The rigidity of the abdominal wall did not allow of any stretching. It was an 8 months' child, passed its natural secretion, fæces, and urine. In the morning it vomited bile, and in the afternoon nursed at its mother's breast; its pulse was regular, as also its respiration. The portions out were made up by the duodenum, jejunum, and ileum of the small intestines, and the colon and a portion of the rectum of the large intestines. It died the next day, the 16th November, having lived exactly 21½ hours.

*Treatment.*—I was called to the aid of the child at 11½ A. M. on the day of its birth. From the time of its birth till then, 2 inches more escaped. By warm and emollient applications, I succeeded in replacing what had, since its birth, escaped (by crying and bearing down); but to crowd in more of the protruded parts was like stuffing a full bag. If the child was handled and the escaped intestines not disturbed, it was quiet; but if they were touched, it would cry. The specimen could not be obtained, nor was a *post mortem* allowed to be made.

*Remarks.*—Similar cases, although of rare occurrence, occasionally occur: a specimen of ectropium of the bladder can be seen in the museum of Chicago Medical College. Judging from the appearance of the fissure, and taking into account the early development of the intestines and abdominal walls, it is very probable that it was a natural defect. At the end of the second month of fœtal gestation, the intestines grow much faster than the abdominal walls; so much so, that they are incapable to hold the mass of the bowels, and they protrude, like a hernia. At this time, the growth of the abdominal walls must have been by some cause arrested in this case, and the above condition made permanent; for it was absolutely impossible to find space enough for an insignificant portion of the protruding intestinal mass. Congenital umbilical hernia occurs by an imperfectly-closed umbilicus; but this case was very much different, for the umbilicus was perfectly formed.

## Figures and Tables

**Figure f1:**